# Supplementary material for: Transition to Fast Whole-Body SPECT/CT Bone Imaging: An Assessment of Image Quality
Source: Diagnostics (Basel). 2022 Nov 24;12(12):2938. doi: 10.3390/diagnostics12122938 (PMC9776819; doi:10.3390/diagnostics12122938)
Supplement: Supplementary file 1 [file diagnostics-12-02938-s001.zip › Table S1.pdf]

Table S1. Image quality assessment, *p* value between every two acquisition periods.

| **Image quality (4 iterations)** | | |
|---|---|---|
| Acquisition protocol | | *p*-value |
| 180s | 360s | **0.499** |
| 180s | 480s | **0.007*** |
| 180s | 900s | **< .001*** |
| 180s | 450s | **0.027*** |
| 360s | 480s | **0.194** |
| 360s | 900s | **0.011*** |
| 360s | 450s | **0.562** |
| 480s | 900s | **0.799** |
| 480s | 450s | **0.862** |
| 900s | 450s | **0.097** |
| **Image quality (8 iterations)** | | |
| Acquisition protocol | | *p*-value |
| 180s | 360s | **0.212** |
| 180s | 480s | **0.001*** |
| 180s | 900s | **< .001*** |
| 180s | 450s | **0.001*** |
| 360s | 480s | **0.036*** |
| 360s | 900s | **0.001*** |
| 360s | 450s | **0.091** |
| 480s | 900s | **0.862** |
| 480s | 450s | **0.827** |
| 900s | 450s | **0.184** |
| **Image quality (12 iterations)** | | |
| Acquisition protocol | | *p*-value |
| 180s | 360s | **0.250** |
| 180s | 480s | **0.041*** |
| 180s | 900s | **0.001*** |
| 180s | 450s | **0.010*** |
| 360s | 480s | **0.631** |
| 360s | 900s | **0.005*** |
| 360s | 450s | **0.124** |
| 480s | 900s | **0.182** |
| 480s | 450s | **0.840** |
| 900s | 450s | **0.556** |
| **Image quality (16 iterations)** | | |
| Acquisition protocol | | *p*-value |
| 180s | 360s | **0.774** |
| 180s | 480s | **0.028*** |
| 180s | 900s | **0.014*** |
| 180s | 450s | **0.028*** |
| 360s | 480s | **0.602** |
| 360s | 900s | **0.054*** |
| 360s | 450s | **0.602** |
| 480s | 900s | **0.731** |
| 480s | 450s | **1.000** |
| 900s | 450s | **0.731** |
