# Supplementary material for: Transition to Fast Whole-Body SPECT/CT Bone Imaging: An Assessment of Image Quality
Source: Diagnostics (Basel). 2022 Nov 24;12(12):2938. doi: 10.3390/diagnostics12122938 (PMC9776819; doi:10.3390/diagnostics12122938)
Supplement: Supplementary file 1 [file diagnostics-12-02938-s001.zip › Table S2.pdf]

Table S2. Image noise assessment, P value between every two acquisition periods.

**Image noise (4 iterations)**		
Acquisition protocol		p-value
180s	360s	**0.958**
180s	480s	**0.011***
180s	900s	**< .001***
180s	450s	**0.039***
360s	480s	**0.042***
360s	900s	**0.001***
360s	450s	**0.110**
480s	900s	**0.012***
480s	450s	**0.930**
900s	450s	**0.0.004***
**Image noise (8 iterations)**		
Acquisition protocol		p-value
180s	360s	**0.130**
180s	480s	**< .001***
180s	900s	**< .001***
180s	450s	**< .001***
360s	480s	**0.238**
360s	900s	**0.021***
360s	450s	**0.317**
480s	900s	**0.429**
480s	450s	**1.00**
900s	450s	**0.542**
**Image noise (12 iterations)**		
Acquisition protocol		p-value
180s	360s	**0.143**
180s	480s	**0.004***
180s	900s	**0.002***
180s	450s	**0.011***
360s	480s	**0.494**
360s	900s	**0.021***
360s	450s	**0.418**
480s	900s	**0.131**
480s	450s	**0.973**
900s	450s	**0.638**
**Image noise (16 iterations)**		
Acquisition protocol		p-value
180s	360s	**0.615**
180s	480s	**0.009***
180s	900s	**0.002***
180s	450s	**0.010***
360s	480s	**0.721**
360s	900s	**0.050***
360s	450s	**0.614**
480s	900s	**0.143**
480s	450s	**0.998**
900s	450s	**0.320**
