# Supplementary material for: Transition to Fast Whole-Body SPECT/CT Bone Imaging: An Assessment of Image Quality
Source: Diagnostics (Basel). 2022 Nov 24;12(12):2938. doi: 10.3390/diagnostics12122938 (PMC9776819; doi:10.3390/diagnostics12122938)
Supplement: Supplementary file 1 [file diagnostics-12-02938-s001.zip › Table S3.pdf]

Table S3. Diagnostic confidence assessment, P value between every two acquisition periods.

| **Diagnostic confidence (4 iterations)** | | |
|---|---|---|
| Acquisition protocol | | p-value |
| 180s | 360s | **0.467** |
| 180s | 480s | **0.017*** |
| 180s | 900s | **< .001*** |
| 180s | 450s | **0.020*** |
| 360s | 480s | **0.267** |
| 360s | 900s | **0.003*** |
| 360s | 450s | **0.267** |
| 480s | 900s | **0.125** |
| 480s | 450s | **0.997** |
| 900s | 450s | **0.458** |
| **Diagnostic confidence (8 iterations)** | | |
| Acquisition protocol | | p-value |
| 180s | 360s | **0.034*** |
| 180s | 480s | **< .001*** |
| 180s | 900s | **< .001*** |
| 180s | 450s | **< .001*** |
| 360s | 480s | **0.374** |
| 360s | 900s | **0.115** |
| 360s | 450s | **0.376** |
| 480s | 900s | **0.968** |
| 480s | 450s | **0.996** |
| 900s | 450s | **0.777** |
| **Diagnostic confidence (12 iterations)** | | |
| Acquisition protocol | | p-value |
| 180s | 360s | **0.644** |
| 180s | 480s | **0.087** |
| 180s | 900s | **0.002*** |
| 180s | 450s | **0.045*** |
| 360s | 480s | **0.451** |
| 360s | 900s | **0.006*** |
| 360s | 450s | **0.268** |
| 480s | 900s | **0.546** |
| 480s | 450s | **0.999** |
| 900s | 450s | **0.690** |
| **Diagnostic confidence (16 iterations)** | | |
| Acquisition protocol | | p-value |
| 180s | 360s | **0.914** |
| 180s | 480s | **0.036*** |
| 180s | 900s | **0.006*** |
| 180s | 450s | **0.034*** |
| 360s | 480s | **0.394** |
| 360s | 900s | **0.082** |
| 360s | 450s | **0.320** |
| 480s | 900s | **0.798** |
| 480s | 450s | **0.998** |
| 900s | 450s | **0.959** |
