# Supplementary material for: Transition to Fast Whole-Body SPECT/CT Bone Imaging: An Assessment of Image Quality
Source: Diagnostics (Basel). 2022 Nov 24;12(12):2938. doi: 10.3390/diagnostics12122938 (PMC9776819; doi:10.3390/diagnostics12122938)
Supplement: Supplementary file 1 [file diagnostics-12-02938-s001.zip › Table S4.pdf]

Table S4. The results were evaluated individually for each reader comparing iterations numbers (4-16) and corresponding p values in different acquisition time for all three categories (image quality, image noise and diagnostic confidence).

Reader 1	**Reference images 900s**				**Overall average**	***p* value**	**480s**				**Overall average**	***p* value**	**450s**				**Overall average**	***p* value**	**360s**				**Overall average**	***p* value**	**180s**				**Overall average**	***p* value**
	4	8	12	16			4	8	12	16			4	8	12	16			4	8	12	16			4	8	12	16		
Image quality	3.60 ± 0.54	3.20 ± 0.83	2.80 ± 0.44	2.40 ± 0.89	**3.00 ± 0.79**	**0.07**	3.00 ± 0.70	3.00 ± 0.70	2.80 ± 0.44	2.40 ± 0.54	**2.65 ± 0.67**	**0.11**	2.60 ± 0.54	2.40 ± 0.54	2.60 ± 0.54	2.20 ± 0.44	**2.45 ± 0.51**	**0.58**	1.80 ± 0.44	2.00 ± 0.00	2.20 ± 0.44	1.80 ± 0.83	**1.95 ± 0.51**	**0.64**	1.60 ± 0.89	1.40 ± 0.54	1.60 ± 0.54	1.60 ± 0.54	**1.55 ± 0.60**	**0.89**
Image noise	4.00 ± 0.00	3.40 ± 0.89	3.40 ± 0.89	3.20 ± 0.83	**3.50 ± 0.76**	**0.27**	3.20 ± 0.44	3.00 ± 0.00	3.00 ± 0.70	2.80 ± 0.44	**3.00 ± 0.45**	**0.57**	3.00 ± 0.70	3.20 ± 0.44	3.20 ± 0.83	2.60 ± 0.89	**3.00 ± 0.72**	**0.33**	2.00 ± 0.00	2.60 ± 0.54	2.80 ± 0.44	2.20 ± 1.10	**2.40 ± 0.75**	**0.21**	2.00 ± 0.00	1.60 ± 0.54	2.00 ± 0.00	1.80 ± 0.44	**1.85 ± 0.85**	**0.58**
Diagnostic confidence	4.00 ± 0.00	3.40 ± 0.89	3.80 ± 0.44	3.20 ± 0.83	**3.60 ± 0.68**	**0.23**	3.00 ± 0.83	3.20 ± 0.83	3.20 ± 0.83	3.00 ± 0.70	**3.10 ± 0.71**	**0.97**	3.20 ± 0.83	3.20 ± 0.44	3.40 ± 0.89	2.80 ± 1.10	**3.15 ± 0.81**	**0.86**	2.40 ± 0.89	2.60 ± 0.54	2.60 ± 0.54	2.00 ± 1.00	**2.40 ± 0.75**	**0.75**	1.80 ± 1.10	1.60 ± 0.54	2.20 ± 1.10	2.00 ± 0.70	**1.90 ± 0.85**	**0.39**

**Reader 2**	**Reference images 900s**				**Overall average**	**p value**	**480s**				**Overall average**	**p value**	**450s**				**Overall average**	**p value**	**360s**				**Overall average**	**p value**	**180s**				**Overall average**	**p value**
	4	8	12	16			4	8	12	16			4	8	12	16			4	8	12	16			4	8	12	16		
Image quality	3.40 ± 0.36	3.20 ± 0.44	3.00 ± 0.00	3.00 ± 0.00	**3.15 ± 0.30**	**0.19**	3.20 ± 0.83	2.80 ± 0.83	2.60 ± 0.54	2.60 ± 0.89	**2.80 ± 0.76**	**0.32**	3.00 ± 0.70	2.80 ± 0.44	2.60 ± 0.54	2.80 ± 0.44	**2.80 ± 0.52**	**0.66**	2.80 ± 0.83	2.0 ± 0.70	1.80 ± 0.44	2.20 ± 0.83	**2.20 ± 0.76**	**0.17**	1.80 ± 0.83	1.60 ± 0.54	1.40 ± 0.54	1.60 ± 0.54	**1.60 ± 0.59**	**0.57**
Image noise	4.00 ± 0.00	3.80 ± 0.44	3.80 ± 0.44	3.60 ± 0.54	**3.80 ± 0.41**	**0.49**	3.40 ± 0.54	3.20 ± 0.83	2.80 ± 0.44	2.60 ± 0.54	**3.00 ± 0.64**	**0.15**	3.20 ± 0.44	3.10 ± 1.00	3.00 ± 1.00	3.00 ± 0.00	**3.07 ± 0.68**	**0.91**	2.60 ± 0.89	2.20 ± 1.09	2.00 ± 0.70	2.20 ± 0.83	**2.25 ± 0.85**	**0.64**	2.00 ± 0.70	1.60 ± 0.54	1.40 ± 0.54	1.60 ± 0.54	**1.65 ± 0.85**	**0.09**
Diagnostic confidence	3.40 ± 0.54	3.20 ± 0.44	3.00 ± 0.00	3.00 ± 0.00	**3.15 ± 0.36**	**0.19**	3.20 ± 0.44	3.00 ± 0.70	2.60 ± 0.89	2.60 ± 0.54	**2.85 ± 0.67**	**0.39**	3.20 ± 0.83	2.80 ± 0.44	2.60 ± 0.54	3.00 ± 0.00	**2.90 ± 0.55**	**0.46**	2.60 ± 0.54	2.40 ± 0.89	2.00 ± 0.70	2.20 ± 0.83	**2.30 ± 0.73**	**0.45**	2.00 ± 0.54	1.60 ± 0.54	1.40 ± 0.54	1.60 ± 0.54	**1.65 ± 0.58**	**0.22**

The mean value ± SD represents the mean score of 5 patient's image, evaluated individually for each reader.

p* values: Significance value between different numbers of iterations for a given duration of acquis
